# Association between genetic polymorphisms in AURKA (rs2273535 and rs1047972) and breast cancer risk: a meta-analysis involving 37,221 subjects

**DOI:** 10.1186/s12935-014-0091-y

**Published:** 2014-09-05

**Authors:** Zhi-Jun Dai, Hua-Feng Kang, Xi-Jing Wang, Yong-Ping Shao, Shuai Lin, Yang Zhao, Hong-Tao Ren, Wei-Li Min, Meng Wang, Xiao-Xu Liu

**Affiliations:** Department of Oncology, the Second Affiliated Hospital of Xi’an Jiaotong University, Xi’an, 710004 China; Center for Translational Medicine, Frontier Institute of Science and Technology (FIST), Xi’an Jiaotong University, Xi’an, 710049 China

**Keywords:** AURKA, Breast cancer, Polymorphism, Susceptibility, Meta-analysis

## Abstract

**Background:**

Published data on the association between AURKA polymorphisms and breast cancer (BC) risk are inconclusive. This meta-analysis was performed to derive a more precise estimation on the relationship between AURKA polymorphisms (rs2273535 and rs1047972) and BC risk.

**Methods:**

PubMed, Web of Knowledge and Embase were searched for relevant studies. Odds ratios (ORs) with 95% confidence intervals (CIs) were used to estimate the strength of associations. The pooled odds ratios (ORs) with 95% confidence intervals (CIs) were performed for allele contrast genetic model, homozygous genetic model, heterozygote genetic model, dominant model, and recessive model, respectively.

**Results:**

A total of 13 studies (16,349 BC patients and 20,872 case-free controls) were involved in this meta-analysis. Meta-analysis showed that there was significant association between rs2273535 and BC risk in three genetic models in the overall population (A vs. T: OR = 1.08, 95% CI = 1.01–1.15, *P* = 0.02; AA vs. TT: OR = 1.36, 95% CI = 1.06-1.73, *P* < 0.00001; AA vs. TT + TA: OR = 1.15, 95% CI = 1.01-1.31, *P* = 0.04). In the subgroup analysis by ethnicity, the effects remained in Asians (allele contrast genetic model: OR = 1.12, 95% CI = 1.00-1.26, *P* = 0.04 and homozygote comparison: OR = 1.22, 95% CI = 1.06-1.41, *P* = 0.007). However, no genetic models reached statistical association in Cauasians. Rs1047972 polymorphism was associated with BC risk in the overall population based on homozygote comparison (AA vs. GG: OR = 0.81, 95% CI = 0.66-0.99, *P* = 0.04). When stratified by ethnicity, rs1047972 polymorphism had a decreased association with BC risk in Caucasians based on allele contrast genetic model, homozygote comparison, the dominant model and the recessive model. However, there was no association in any genetic model in Asians.

**Conclusions:**

This meta-analysis suggests that AURKA rs2273535 polymorphism has an increased risk with BC, especially in Asians. However, rs1047972 polymorphism has a decreased BC risk in Caucasians. Further large scale multicenter epidemiological studies are warranted to confirm this finding.

## Introduction

Breast cancer (BC) is the most common cancer in women and the incidence has increased in recent years worldwide [1]. BC is also a complex disease with multiple epidemiological, genetic, and epigenetic factors contributing to disease etiology [2,3]. About 10% of all breast cancers are associated with family history [4]. The clinical features of human BC are characterized by a considerable heterogeneity.

AURKA, also known as STK15/Aurora-A, belongs to the Aurora family of cell cycle-regulating serine/threonine kinase [5]. AURKA is localized at the centrosome from the time of centrosome duplication to mitotic exit and regulates centrosome function [6]. AURKA plays an important role in mitotic centrosome separation, maturation and spindle formation and stability [5–7]. Studies have demonstrated that AURKA overexpression contributes to genetic instability and tumourigenesis by disrupting the proper assembly of the mitotic checkpoint complex and occurs in a high proportion of ovarian, bladder, gastric and breast cancers [8–11].

Two nonsynonymous polymorphisms F31I (rs2273535) and V57I (rs1047972) have been identified in the AURKA gene. Both polymorphisms are located within two conserved motifs in the N-terminal region of the AURKA gene [12]. Several studies have reported the role of AURKA polymorphisms in BC risk [13–25], but the results are inconclusive. For example, Ruan et al. [13] reported that the AA (Ile/Ile) genotype of rs2273535 was associated with a significantly increased risk of breast cancer among the Chinese Han population. However, Dai et al. Dai et al. [23] that rs2273535 allele of the AURKA gene was not associated with breast cancer risk (OR 1.2, 95% CI 0.9–1.6). There was also no apparent difference in allele frequency or genotype of rs1047972 polymorphism (OR 0.8, 95% CI 0.4–1.6) [23]. Fletcher et al. [20] demonstrated that the Ile/Ile homozygous genotype was not associated with an increased BC risk in white women of British descent. Egan et al. described an increased risk associated with a compound genotype of the two polymorphisms, rs2273535 and rs1047972, with individuals homozygous for the 31I and 57 V alleles having a nearly 2-fold increase in risk of postmenopausal invasive BC [24].

Therefore, we carried out this meta-analysis on all eligible case–control studies to derive a more precise estimation of the associations between AURKA polymorphisms (rs2273535 and rs1047972) with BC risk.

## Materials and methods

### Publication search

We searched the articles in PubMed, Web of Knowledge and Embase to collect articles with case–control studies related to the association of AURKA polymorphisms and BC risk. The keywords were as follows: breast cancer/breast carcinoma, AURKA/Aurora-A/STK15, polymorphism/genotype/SNP. All qualified studies were searched until May 30, 2014. The eligible articles must be published in English. Furthermore, reference lists of main reports and review articles were also reviewed by a manual search to identify additional relevant publications.

### Selection and exclusion criteria

The following criteria were used to select studies for further meta-analysis: (1) case–control studies; (2) the studies evaluated the associations between AURKA polymorphisms and breast cancer risk; (3) all cases were diagnosed by pathological examination; (4) the studies contained sufficient genotype data for estimating an odds ratio (OR) with 95% confidence interval (CI); (5) genotype distributions of controls passed Hardy-Weinberg equilibrium (HWE) test.

Accordingly, the following exclusion criteria were also used: (1) the design of the experiments were not case–control studies; (2) the source of cases and controls, and other essential information were not provided; (3) the genotype distribution of the control population was departure from HWE; (4) reviews, meta-analysis and duplicated publications.

### Data extraction and synthesis

Information was carefully extracted from all eligible studies independently by two authors according to the inclusion criteria listed above. In case of disagreements, another author was consulted to resolve the dispute, and a final decision was made by the majority of the votes. For each included study, the following information was collected: first author, year of publication, country of origin, ethnicity, source of control, numble of cases and controls, genotype methods, allele and genotype frequencies and evidence of HWE in controls. Different ethnicity descents were categorized as Caucasian, Asian, and “mixed”. All the case and control groups were well controlled. The non-cancer controls had no history of gynecologic disease, and there was no present evidence of any malignant disease.

### Statistical analysis

The associations between AURKA polymorphisms and BC risk were measured by odds ratio (OR) with 95% confidence interval (CI). The significance of the pooled OR was determined by the Z test. Statistical heterogeneity among studies was assessed with the Q and I^2^ statistics. If the *P* value of heterogeneity test was more than 0.1 (*P* ≥ 0.1), the pooled OR estimate of the study was calculated by the fixed-effects model. Otherwise, the random-effects model was used [26]. The value of the I index is used to assess the degree of heterogeneity (I^2^ < 25%: no heterogeneity; 25% < I^2^ < 50%: moderate heterogeneity; 50% < I^2^ < 75%: high heterogeneity; I^2^ > 75%:extreme high heterogeneity). Publication bias were evaluated by the funnel plot and further assessed by the method of Egger’s linear regression test. All statistical analyses were carried out with the review manager version 5.1 (Revman; The Cochrane Collaboration, Oxford, UK).

## Results

### Characteristics of eligible studies

**Figure 1:**
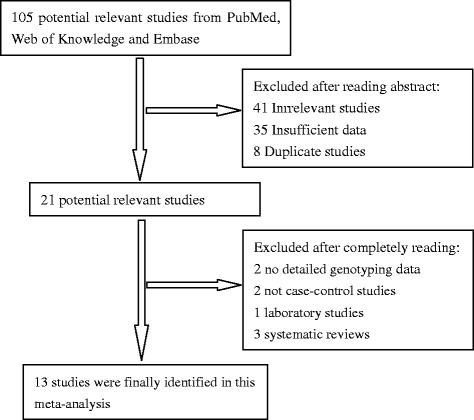
**Flow chart of studies selection in this meta-analysis.**

As shown in Figure 1, a total of 105 potential publications were extracted at first based on our selection strategy. According to the inclusion criteria defined above, 13 studies on AURKA polymorphisms and BC risk were finally identified in this meta-analysis [13–25]. These studies included 16,349 BC patients and 20,872 cancer-free controls. The characteristics of the included studies are listed in Tables 1. Among the eligible thirteen studies, seven studies were performed in Caucasians from Sweden, Germany, Iceland, UK and USA [14,15,17–20,24]. Four were based on Asian background and were carried out in China [13,22,23,25]. Two studies were mixed ethnicity descent [16,21]. All studies were case–control studies. All BC cases were confirmed by histology or pathology. Moreover, controls were mainly matched on age, of which nine were population-based and two were hospital-based studies.

**Table 1:** **Characteristics of the studies included in the meta-analysis**

**First author**	**Year**	**Country**	**Ethnicity**	**Design**	**Genotyping medthod**	**Number(case/control)**	**SNP**	**HWE**
Ruan [13]	2011	China	Asian	PB	TaqMan	1334/1568	rs2273535	0.76
Shi [14]	2011	Sweden	Caucasian	PB	TaqMan	763/1516	rs2273535	0.23
MARIE-GENICA [15]	2010	German	Caucasian	PB	MALDI-TOF	3137/5469	rs2273535	0.12
							rs1047972	0.17
Couch [16]	2007	CIMBA	Mixed	PB	TaqMan	3884/3303	rs2273535	NA
Tchatchou [17]	2007	German	Caucasian	PB	TaqMan	727/819	rs2273535	0.60
Vidarsdottir [18]	2007	Iceland	Caucasian	NA	PCR-RFLP	759/653	rs2273535	0.07
Cox [19]	2006	USA	Caucasian	NA	TaqMan	1259/1742	rs2273535	0.31
							rs1047972	0.70
Fletcher [20]	2006	UK	Caucasian	PB	PCR-RFLP	507/875	rs2273535	0.13
Ewart-Toland [21]	2005	USA	Mixed	PB	SnAPSHOT	898/448	rs2273535	0.81
Lo [22]	2005	China(Taiwan)	Asian	HB	TaqMan	709/1972	rs2273535	0.23
							rs1047972	0.80
Dai [23]	2004	China	Asian	PB	TaqMan	1102/1188	rs2273535	0.07
							rs1047972	0.68
Egan [24]	2004	USA	Caucasian	PB	Sequencing	941/830	rs2273535	0.31
							rs1047972	0.68
Sun [25]	2004	China	Asian	HB	PCR-RFLP	520/520	rs2273535	0.11

HWE: Hardy–Weinberg equilibrium; PB: population based; HB: hospital-based; NA: not available; PCR: polymerase chain reaction; RFLP: restriction fragment length polymorphism; MALDI–TOF MS: Matrix-Assisted Laser Desorption/Ionization Time of Flight Mass Spectrometry; CIMBA: the Consortium of Investigators of Modifiers of BRCA1/2 including 16 clinic and population-based research studies and multicenter consortia.

### Meta-analysis results

As shown in Table 2, the frequencies of the minor allele in BC patients varied widely across the eligible studies, ranging from 0.19 to 0.82 (rs2273535), 0.13 to 0.16 (rs1047972). The average frequencies of the minor allele in the two polymorphisms were 0.39 and 0.15, respectively.

**Table 2:** **AURKA polymorphisms genotype distribution and allele frequency in cases and controls**

**First author**	**Genotype (N)**								**Allele frequency (N)**				**MAF (Case/Control)**
	**Case**				**Control**				**Case**		**Control**		
	**Total**	**AA**	**Aa**	**aa**	**Total**	**AA**	**Aa**	**aa**	**A**	**a**	**A**	**a**	
rs2273535 (T > A)													
Ruan 2011	1334	599	568	167	1568	716	691	161	1766	902	2123	1013	0.34/0.32
Shi 2011	763	27	222	514	1516	71	478	967	276	1250	620	2412	0.82/0.80
MARIE-GENICA 2010	3076	1873	1096	107	5466	3290	1927	249	4842	1310	8507	2425	0.21/0.22
Couch 2007	3884	3696		188	3303	3128		175	-	-	-	-	-
Tchatchou 2007	727	37	257	433	819	47	287	485	331	1123	381	1257	0.77/0.77
Vidarsdottir 2007	759	429	288	42	653	401	231	21	1146	372	1033	273	0.25/0.21
Cox 2006	1241	774	401	66	1711	1075	571	65	1949	533	2721	701	0.22/0.21
Fletcher 2006	507	335	154	18	875	547	280	48	824	190	1374	376	0.19/0.22
Ewart-Toland 2005	898	533	303	62	448	279	148	21	1369	427	706	190	0.24/0.21
Lo 2005	707	71	288	348	1969	196	887	886	430	984	1279	2659	0.70/0.68
Dai 2004	1102	121	491	490	1186	149	503	534	733	1471	801	1571	0.67/0.66
Egan 2004	940	559	331	50	830	516	283	31	1449	431	1315	345	0.23/0.21
Sun 2004	520	50	214	256	520	66	262	192	314	726	394	646	0.70/0.62
rs1047972 (G > A)													
MARIE-GENICA 2010	3139	2220	850	69	5469	3737	1561	171	5290	988	9035	1903	0.16/0.17
Cox 2006	1240	870	342	28	1724	1215	462	47	2082	398	2892	556	0.16/0.16
Lo 2005	704	543	146	15	1950	1506	414	30	1232	176	3426	474	0.13/0.12
Dai 2004	1102	805	281	16	1188	908	263	17	1891	313	2079	297	0.14/0.13
Egan 2004	905	637	245	23	788	542	225	21	1519	291	1309	267	0.16/0.17

A represents the major allele, a represents the minor allele. MAF: minor allele frequencies.

**Figure 2:**
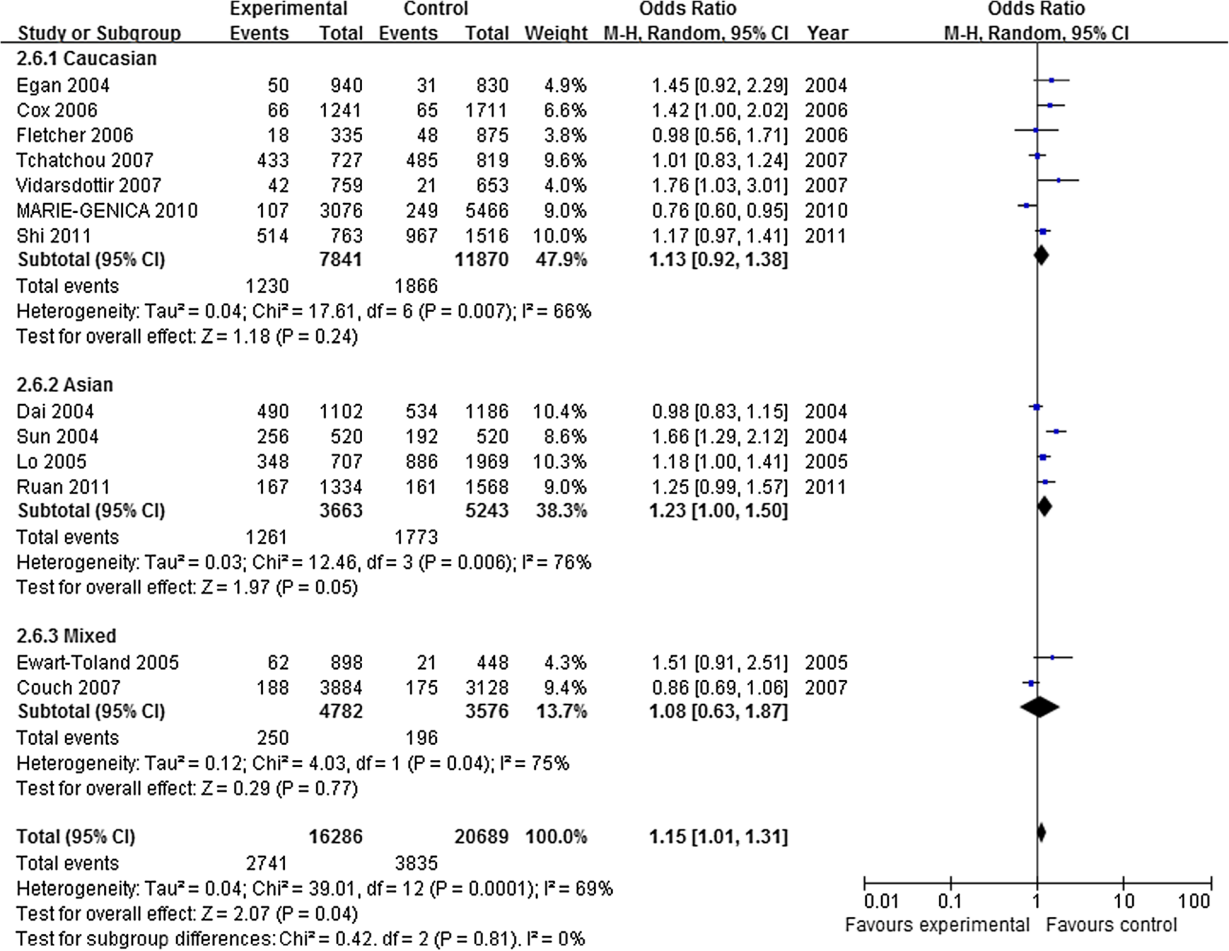
**Forest plots of AURKA rs2273535 polymorphism and BC risk (AA vs TT + TA).** The squares and horizontal lines correspond to the study specific OR and 95% CI. The area of the squares reflects the weight (inverse of the variance). The diamond represents the summary OR and 95% CI.

The main results of this meta-analysis were listed in Table 3. There were 13 studies with 16,286 BC patients and 20,689 case-free controls for AURKA rs2273535 polymorphism. As show in Table 3 and Figure 2, there was significant association between rs2273535 and BC risk in three genetic models in the overall population (A vs. T: OR = 1.08, 95% CI = 1.01–1.15, *P* =0.02; AA vs. TT: OR = 1.36, 95% CI = 1.06-1.73, *P* < 0.00001; AA vs. TT + TA: OR = 1.15, 95% CI = 1.01-1.31, *P* = 0.04), while there was no significant association in heterozygote comparison and the dominant model (TA vs. TT: OR =1.02, 95% CI = 0.96-1.08, *P* = 0.52; TA + AA vs. TT: OR = 1.04, 95% CI = 0.98-1.09, *P* = 0.20). In the subgroup analysis by ethnicity, the effects remained in Asians (allele contrast genetic model: OR = 1.12, 95% CI = 1.00-1.26, *P* = 0.04 and homozygote comparison: OR = 1.22, 95% CI = 1.06-1.41, *P* = 0.007). However, no genetic models reached statistical association in Cauasians (Table 3).

**Table 3:** **Meta-analysis results**

**Comparisons**	**OR**	**95%CI**	***P*** **value**	**Heterogeneity**		**Effects model**
				**I** ^**2**^	***P*** **value**	
a vs A						
rs2273535	1.08	1.01–1.15	0.02	62%	0.002	R
Caucasian	1.05	0.96–1.14	0.31	63%	0.01	R
Asian	1.12	1.00–1.26	0.04	66%	0.03	R
rs1047972	0.98	0.89–1.08	0.75	55%	0.06	R
Caucasian	0.92	0.86–0.98	0.01	0%	0.38	F
Asian	1.10	0.97–1.25	0.14	0%	0.37	F
aa vs AA						
rs2273535	1.36	1.06–1.73	<0.00001	82%	<0.00001	R
Caucasian	1.32	0.85–2.03	0.21	88%	<0.00001	R
Asian	1.22	1.06–1.41	0.007	24%	0.27	F
rs1047972	0.81	0.66–0.99	0.04	22%	0.28	F
Caucasian	0.74	0.59–0.93	0.009	0%	0.56	F
Asian	1.22	0.77–1.95	0.40	0%	0.57	F
Aa vs AA						
rs2273535	1.02	0.96–1.08	0.52	0%	0.83	F
Caucasian	1.02	0.95–1.09	0.64	0%	0.67	F
Asian	1.02	0.90–1.14	0.80	0%	0.49	F
rs1047972	0.98	0.91–1.05	0.51	41%	0.14	F
Caucasian	0.94	0.87–1.02	0.15	0%	0.46	F
Asian	1.10	0.95–1.26	0.20	51%	0.15	F
Aa + aa vs AA						
rs2273535	1.04	0.98–1.09	0.20	12%	0.33	F
Caucasian	1.02	0.95–1.08	0.61	31%	0.19	F
Asian	1.07	0.96–1.20	0.21	0%	0.48	F
rs1047972	0.99	0.89–1.10	0.84	51%	0.09	R
Caucasian	0.92	0.86–1.00	0.04	0%	0.40	F
Asian	1.10	0.96–1.27	0.16	33%	0.22	F
aa vs AA + Aa						
rs2273535	1.15	1.01–1.31	0.04	69%	0.0001	R
Caucasian	1.13	0.92–1.38	0.24	66%	0.007	R
Asian	1.23	1.10–1.50	0.05	76%	0.006	R
rs1047972	0.82	0.67–1.00	0.05	14%	0.33	F
Caucasian	0.75	0.60–0.94	0.01	0%	0.60	F
Asian	1.20	0.75–1.91	0.44	0%	0.50	F

A: represents the major allele, a: represents the minor allele, F: fixed effects model, R: random effects model.

**Figure 3:**
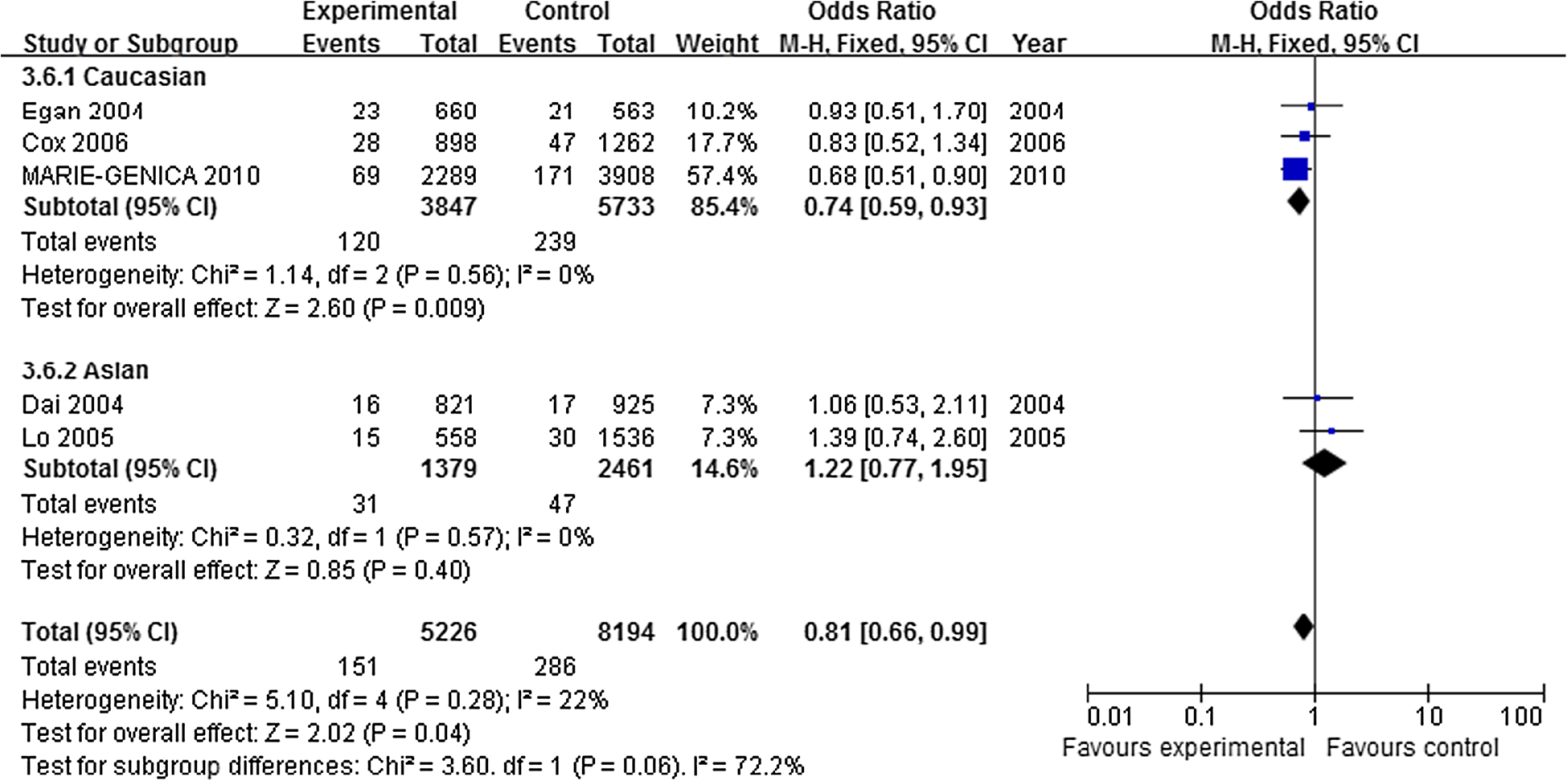
**Forest plots of AURKA rs1047972 polymorphism and BC risk (AA vs GG).** The squares and horizontal lines correspond to the study specific OR and 95% CI. The area of the squares reflects the weight (inverse of the variance). The diamond represents the summary OR and 95% CI.

5 studies with 7,090 cases and 11,119 controls were used to assess the relationship between rs1047972 polymorphism and BC risk. As shown in Table 3 and Figure 3, rs1047972 polymorphism was associated with BC risk in the overall population based on homozygote comparison (AA vs. GG: OR = 0.81, 95% CI = 0.66-0.99, *P* = 0.04). There was no association in these four genetic models (allele contrast genetic model: OR = 0.98, 95%CI = 0.89-1.08, *P* = 0.75; heterozygote comparison: OR =0.98, 95% CI = 0.91-1.05, *P* = 0.51; dominant model: OR =0.99, 95% CI = 0.89-1.10, *P* = 0.84); recessive model (OR = 0.82, 95% CI = 0.67-1.00, *P* = 0.05). When stratified by ethnicity, rs1047972 polymorphism had a decreased association with BC risk in Caucasians based on allele contrast genetic model: OR = 0.92, 95%CI = 0.86-0.98, *P* = 0.01; homozygote comparison: OR = 0.74, 95% CI = 0.59-0.93, *P* = 0.009; dominant model: OR =0.92, 95% CI = 0.86-1.00, *P* = 0.04); recessive model (OR = 0.75, 95% CI = 0.60-0.94, *P* = 0.01). However, there was no association in any genetic model in Asians.

### Publication bias

**Figure 4:**
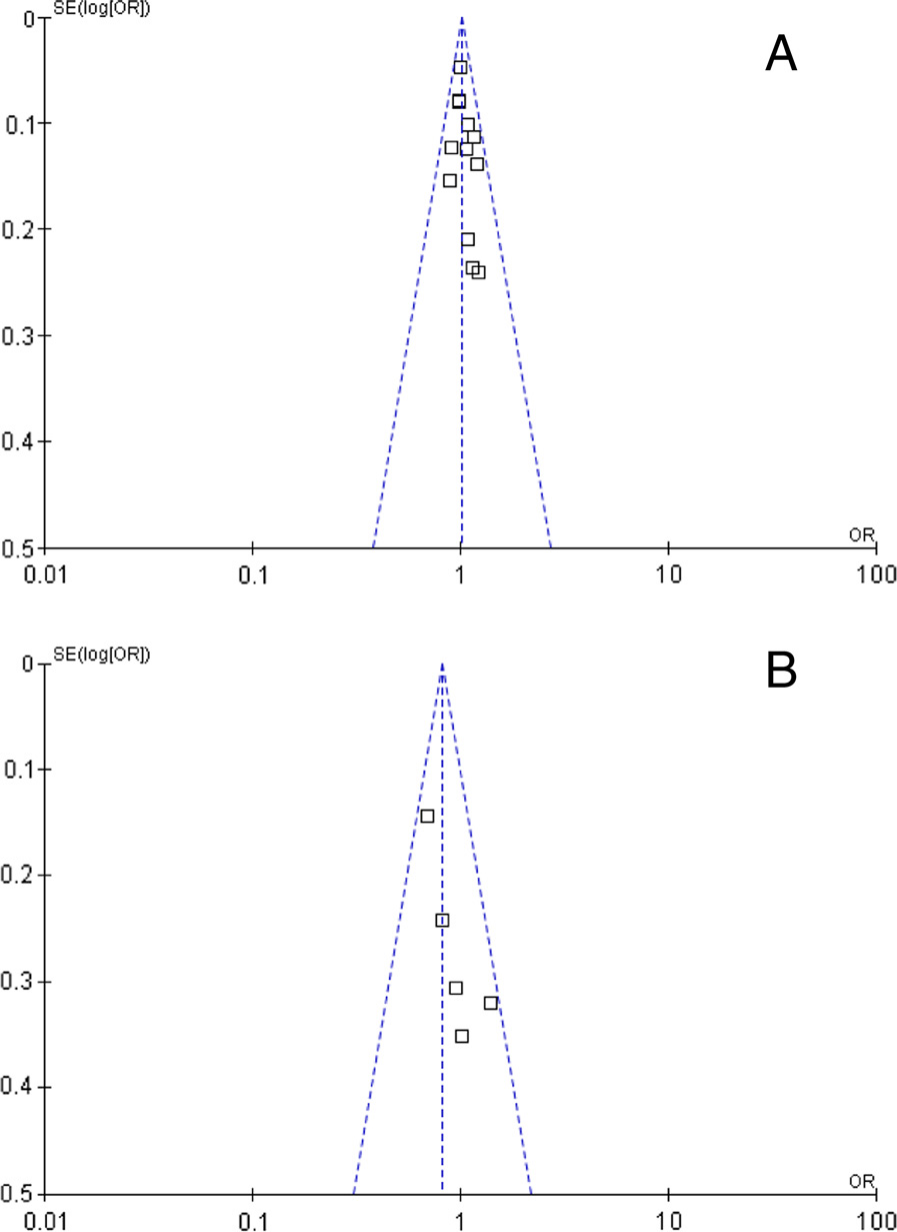
**Funnel plot assessing evidence of publication bias from the eligible studies. A**: F31I (rs2273535); **B**: V57I (rs1047972).

Begg's funnel plot and Egger's test were performed to assess the publication bias. As shown in Figure 4, the funnel plots did not reveal any obvious asymmetry in all genotypes in overall population, and the results of Egger’s test revealed no publication bias (*P* > 0.05).

## Discussion

AURKA is associated with centrosomes, being localized at the centrosome just prior to the onset of mitosis and for the duration of mitosis. Overexpression of AURKA leads to centrosome amplification and cellular transformation [27]. This threonine kinase belongs to a family of mitotic kinases that maintain chromosomal stability through phosphorylation. Thus, any severe defects in AURKA, such as mutations, would lead to drastic genomic instability and induce apoptosis through cell cycle checkpoint surveillance [11,28]. Consequently, the cell harboring a defective AURKA may lead to cancer [11].

Epidemiological studies have been performed to assess the association of AURKA polymorphisms (rs2273535 and rs1047972) with BC risk. However, the results are conflicting. Thus, we conducted a comprehensive meta-analysis involving published data, to assess the strength of association between the two polymorphisms and BC risk. In this present meta-analysis, 13 studies with 16,286 BC patients and 20,689 case-free controls concerning the rs2273535 polymorphism, 5 studies with 7,090 cases and 11,119 controls concerning the rs1047972 polymorphism, were included, respectively. And we explored the association between the two potentially functional polymorphisms of AURKA and BC risk.

In the overall population, we found that rs2273535 polymorphism had an increased association with BC risk in three genetic models (allele contrast genetic model: OR = 1.08, 95% CI = 1.01–1.15, *P* =0.02; homozygote comparison: OR = 1.36, 95% CI = 1.06-1.73, *P* < 0.00001; recessive model: OR = 1.15, 95% CI = 1.01-1.31, *P* = 0.04). However, rs1047972 polymorphism had a decreased association with BC risk based on homozygote comparison (OR = 0.81, 95% CI = 0.66-0.99, *P* = 0.04).

In the stratified analysis based on ethnicity, rs2273535 polymorphism had increased BC risk in Asians based on allele contrast genetic model, homozygote comparison, and the recessive model. However, no genetic models reached statistical association in Cauasians. For rs1047972 polymorphism, there was no significant association in Asians. Rs1047972 had a decreased BC risk in Caucasians based on allele contrast genetic model, homozygote comparison, the dominant model, and the recessive model.

In a previous meta-analysis by Qin et al. [29], they failed to find any significant association between rs1047972 polymorphism and BC risk. It is worth mentioning, the results of the present study are not in accordance with Qin’s analysis. This discrepancy may result from different sample size and ethnic groups. There were only seven studies including 5966 cases and 7609 controls in Qin’s meta-analysis [29], while 13 studies (16,349 BC patients and 20,872 case-free controls) were involved in our meta-analysis.

There are some limitations in this meta-analysis. Firstly, this meta-analysis was based on pooled data. We could not assess the risk of cancer according to stratification of age, smoking, alcohol consumption, environment factors, and other risk factors. Secondly, no individual data such as pathological type, histologic grade, and other clinicopathological index was available. Thus, we could not assess the clinicopathological significance of the polymorphisms in BC. Thirdly, there were only published studies including in the meta-analysis. It is possible that some related unpublished studies that might meet the inclusion criteria were missed. Finally, the included studies were mainly based on Caucasian background. There were only four studies based on Asian background and none based on African background. Further large scale multicenter studies based on Asian or African are warranted to further validate on AURKA polymorphisms and BC risk.

## Conclusion

In summary, this meta-analysis suggests that AURKA rs2273535 polymorphism has an increased risk with BC, especially in Asians. However, rs1047972 polymorphism has a decreased BC risk in Caucasians. Our results indicate that AURKA rs2273535 is a candidate gene polymorphism, and rs1047972 polymorphism is a protective factor for BC cancer risk. We conclude that the two polymorphisms may be potential biomarkers in diagnosis and prediction of BC risk. Further large scale epidemiological studies are needed to confirm these findings.
